# Effects of food quantity and quality on the life history of *Daphnia lumholtzi* in Mwanza Gulf (Lake Victoria, Tanzania)

**DOI:** 10.1093/plankt/fbaf042

**Published:** 2025-08-30

**Authors:** Ilse J M Cornelissen, Jacobus Vijverberg, Theo H Frank, Leopold A J Nagelkerke

**Affiliations:** Netherlands Institute of Ecology (NIOO-KNAW), Droevendaalsesteeg 10, Wageningen 6708 PB, the Netherlands; Wageningen Universiy & Research, Aquaculture and Fisheries Group, De Elst 1, Wageningen 6708 WD, the Netherlands; Netherlands Institute of Ecology (NIOO-KNAW), Droevendaalsesteeg 10, Wageningen 6708 PB, the Netherlands; Netherlands Institute of Ecology (NIOO-KNAW), Droevendaalsesteeg 10, Wageningen 6708 PB, the Netherlands; Marwei 104, 8508 RH Delfstrahuizen, the Netherlands; Wageningen Universiy & Research, Aquaculture and Fisheries Group, De Elst 1, Wageningen 6708 WD, the Netherlands

**Keywords:** cladocerans, Cyanobacteria, food-web interactions, tropical lakes, zooplankton

## Abstract

Until the 1950s, large-bodied calanoids and cladocerans dominated the zooplankton community of Lake Victoria, whereas cyclopoid copepods only comprised 10% of microcrustaceans. From the 1960’s onwards, cyclopoid copepods increased to 70–90% of zooplankton and cladocerans, now dominated by small species, decreased to ca. 5%. Concomitantly phytoplankton biomass increased and shifted from dominance of diatoms to Cyanobacteria, which were hypothesized to be of less nutritional quality, causing the shift in zooplankton. We investigated whether the natural assemblage of Cyanobacteria in Mwanza Gulf negatively affected growth and fecundity of cladocerans. In 2010–2011, we performed life-history experiments with the cladoceran *Daphnia lumholtzi*, feeding it natural seston from Mwanza Gulf from three different locations. A laboratory-strain of the green alga *Scenedesmus obliquus*, proven to be high-quality food, was used as a control. Growth of *D. lumholtzi* in the rainy season and at one station in the dry season was just as high as in the control treatment. If there were negative effects of natural seston these were small. Although the evidence is circumstantial, this suggests that Cyanobacteria and/or their detritus could have been better food than expected and that food quality is not limiting the growth of *D. lumholtzi* in *L. Victoria*.

## INTRODUCTION

Lake Victoria, the largest tropical lake in the world with a surface area of 68 800 km^2^ (Fryer and Iles, 1972), has historically supported one of the most diverse fish communities among the African Great Lakes. During the early 20th Century, the fish community was dominated by > 500 species of haplochromine cichlids (Cichlidae, Haplochromini: Witte and van Oijen, 1990) and large fish species, such as the endemic tilapiines (Cichlidae, Tilapiini), catfishes (Siluriformes), marbled lungfish (*Protopterus aethiopicus*; Heckel, 1851), and freshwater elephantfishes (Mormyridae) (Witte and van Densen, 1995).

Although eutrophication and food-web changes caused by increased human influences were already noticeable from the 1920s onwards (Hecky, 1993; Njagi *et al.,* 2022), the most dramatic changes were observed at the beginning of the 1980s. Then the Nile perch, *Lates niloticus* (Linnaeus, 1758) population expanded, haplochromines were decimated, and the remaining large native fishes declined. The lake became eutrophic, and the fishery became dominated by three species only: two non-native species, Nile perch and Nile tilapia, *Oreochromis niloticus* (Linnaeus, 1758), and the indigenous pelagic cyprinid, *Rastrineobola argentea* (Pellegrin, 1904)*,* known as dagaa in Swahili (Witte *et al.,* 1992; Hecky, 1993).

As for historical developments in Lake Victoria’s zooplankton, the first quantified and well-defined samples were taken in 1927 (Worthington, 1931). Thirteen samples were collected by vertical plankton net hauls every three hours from a depth of 67 m to the surface. However, all samples were taken at the same location (“...near the northeast end of the lake, but some 30 miles from the shore.”: Worthington, 1931) and therefore cannot be considered representative for Lake Victoria as a whole. All other samples, taken between 1888 and 1931, were neither quantified, nor is it known how and where they were taken, or how they were counted (Rzóska, 1957).

Rzóska (1957) attempted to establish an adequate basis for studies on the crustacean zooplankton of Lake Victoria (cladocerans and copepods). A brief survey was carried out in April 1956 with the assistance of the East African Fisheries Research Institute at Jinja (Uganda). He re-examined previous literature, analyzed the zooplankton composition of a series of samples taken in 1950 by Dr Lind (Makerere College) in bays and gulfs of the lake, and took ten new samples, nine from Pilkington Bay and one offshore from Jinja. All samples were collected with a plankton net from the surface during the day. All samples were dominated by copepods, although the densities and sizes of cladocerans were likely underestimated, as they usually show vertical migration behavior, staying deeper in the water column during daytime, with larger species migrating to greater depths than smaller species (Ringelberg, 1999). Cladoceran densities at the inshore stations were lower (3–12% of the total densities) than at the offshore stations (28–37%) (Rzóska, 1957). Worthington (1931) found 39% cladocerans in 1927 offshore, which was not much higher. However, the size distributions were different, with more larger species such as *Daphnia* spp. in 1927 than in 1956, which could be partly explained by different collection methods.

When Akiyama *et al.* (1977) sampled Mwanza Gulf at one station at monthly intervals from April 1973 to January 1975, the strong copepod dominance at inshore stations was confirmed, although there had been a switch towards higher relative densities of small-sized cyclopoid copepods, with a decrease of large-sized calanoid copepods. However, the relative density of cladocerans remained relatively constant around 5% of the total microcrustacean density, mostly consisting of small species (Akiyama *et al.,* 1977; Wanink *et al.,* 2002). The dominance of cyclopoid copepods in Mwanza Gulf has since been confirmed by Waya and Mwambungu (2004), who reported 72% cyclopoid copepods and 6% cladocerans, and by previously unpublished data from Mwanza Gulf 2009–2011 (Suppl. material Table S1), with 78% cyclopoid copepods and 5% cladocerans, the latter being dominated by the small cladoceran *Moina micrura* (45%) and *Bosmina longirostris* (29%).

Some authors suggested that the shift from large herbivorous cladocerans and calanoid copepods to small-bodied predatory cyclopoids was caused by the increase of the zooplanktivorous cyprinid dagaa (*R. argentea*), which rose concomitantly with Nile perch (Gophen *et al.,* 1995). In contrast, Wanink *et al.* (2002) argued that this is not likely since the changes in relative densities of calanoids and cladocerans already started before the population increase of dagaa.

Increased eutrophication caused by a growing human population around the lake already occurred from the 1920s onwards (Hecky, 1993; Njagi *et al.,* 2022). As a result, phytoplankton biomass increased and the community shifted from a dominance of diatoms towards Cyanobacteria (Hecky, 1993; Mugidde, 1993; Sitoki *et al.,* 2010). This may have resulted in toxicity and nutritional inadequacy of the algal food, causing the observed shift in zooplankton composition.

Nutritional quality of Cyanobacteria is generally known to be poor for zooplankton (Gulati and DeMott, 1997). Most zooplankton species grow slower, reproduce less, and generally have higher mortality when only fed Cyanobacteria, compared to a diet also including other phytoplankton taxa such as cryptophytes and green algae (Tillmanns *et al.,* 2008). These nutritional effects primarily result from mismatches between the lipid content of Cyanobacteria and their zooplankton consumers (Von Elert *et al.,* 2003).

Prey selection is the key trait for tolerating cyanobacterial blooms for many zooplankton species. Grazing selection is the ability to graze on nutritious prey while avoiding ingestion of harmful particles. Copepods are highly selective (DeMott, 1986) and can actively detect and avoid ingesting Cyanobacteria from mixed prey (DeMott and Moxter, 1991), and often co-exist with high densities of Cyanobacteria (Bouvy *et al.,* 2001; Koski *et al.,* 2002). In contrast, cladocerans are generalist grazers, with little ability to handle individual food particles (Kirk and Gilbert, 1992), which may lead to inhibited feeding during cyanobacterial blooms (e.g. Lürling and Van Der Grinten, 2003).

In this study we investigate the hypothesis that the assemblage of Cyanobacteria in the Mwanza Gulf negatively affects the growth and fecundity of cladocerans, resulting in low population growth, which can ultimately have been one of the causes for the observed changes in Lake Victoria’s zooplankton community over time. We tested this by running two life-history experiments in the laboratory using *Daphnia lumholtzi* G.O. Sars, 1885 as experimental animal. One experiment was performed in August/September 2010 (dry season) and one in April 2011 (rainy season). As food we used natural seston of the Mwanza Gulf from three different stations, along a eutrophication gradient. We used a laboratory strain of the green algae *Scenedesmus obliquus* as a control to test for nutritional inadequacy of the natural seston.

## MATERIALS AND METHODS

### Study area

**Fig. 1:**
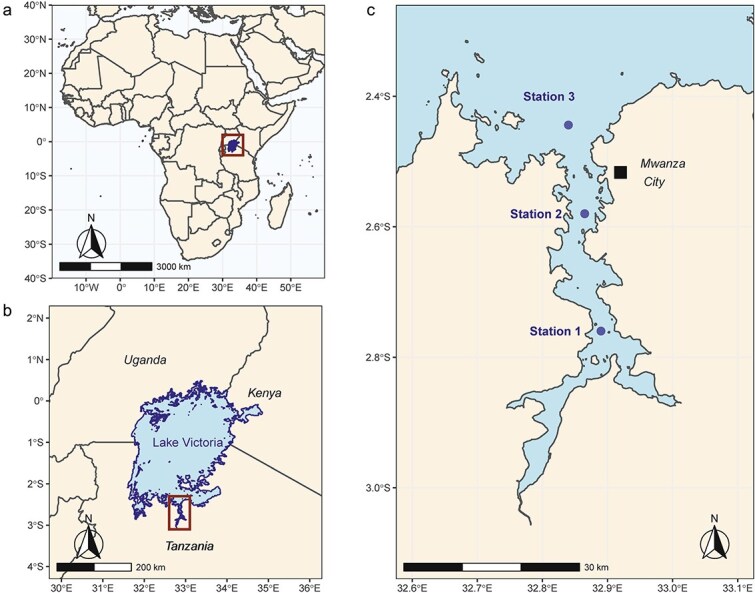
Location of Lake Victoria in Africa (a, b) and the three sampling stations (stations 1, 2 and 3) in Mwanza Gulf (c). Station 1 has a depth range of < 5 m, station 2 a depth range of 6–8 m and station 3 a depth range of 25–28 m.

Mwanza Gulf is located in southern Lake Victoria in Tanzania and is about 60 km long, 2.5–11 km wide, 3–25 m in depth, and covers a surface area of ~500 km^2^ (Witte and van Densen, 1995). Mwanza City is situated in the north near the entrance of the Gulf (Fig. 1); here the shoreline is completely urbanized. Outside of the city the littoral zone is characterized by a mixed vegetation of papyrus, reeds and water hyacinth and by rock formations. The south of the Gulf at station 1 is shallow with a depth of < 5 m. This area can be considered as a littoral habitat with benthic and terrestrial influences. Station 3 is in the open lake outside the Gulf with a depth of 25–28 m. This area can be considered as a pelagic habitat. Station 2 has an intermediate depth range of 6–8 m with both littoral and pelagic habitat characteristics. At each station a pooled sample of 2 × 3 samples were taken with a Van Dorn water sampler at three different depths: just below the surface, at the euphotic depth and at intermediate depth. Since stations showed different depths sampling depths also differed. For station 1 sampling depths were at the surface, at 2.5, and at 4 m depth, for station 2: surface, 3 and 6 m depth, and for station 3: surface, 5 and 10 m. Immediately after return to the laboratory chlorophyll content was measured with a Hydrolab probe.

Climate in the Lake Victoria area is characterized by three seasons based on the yearly monsoon cycles. During the cooler dry season, from June to August, strong southerly winds and low precipitation keep the water well-mixed, isothermal and oxygenated (Talling, 1966). During the short rainy season, from September to December, winds decline and rainfall and temperature increase, warming up the surface layer of the water column, which becomes gradually stratified. During the long rainy season, from January to May, precipitation is high, and stratification of the water column becomes strong (Talling, 1966).

We sampled the phytoplankton community of Mwanza Gulf during the dry and rainy season in 2010–2011, simultaneously and at the same stations as the seston for the present experimental life-history study. These results are presented in Frank *et al.* (2023). In Mwanza Gulf Cyanobacteria dominated during both dry and rainy seasons at all stations (76–95%), followed by Chlorophyta, whereas the contribution of Bacillariophyceae was insignificant (Frank *et al.,* 2023). Phytoplankton was dominated by small cyanobacterial colonies with cell sizes of 0.2–3.0 μm, e.g. *Aphanocapsa* spp., *Aphanothece, Merismopedia* spp. embedded in mucus in both rainy and dry seasons (Table I) (Frank *et al.,* 2023). The filamentous Cyanobacteria *Planktolyngbya* spp. formed the second dominant group of Cyanobacteria in Mwanza Gulf (Table I). Of these groups of cyanobacteria, several abundant species occurring in the Mwanza Gulf are potentially toxic (Jakubowska *et al.,* 2013, Jakubowska and Szeląg-Wasielewska, 2015).

**Table I:** Abundance of the five most abundant phytoplankton genera (all Cyanobacteria) in Mwanza Gulf (2010–2011), showing their mean relative abundance (%) partially based on Frank *et al.* (2023)

Phytoplankton genus	Mean relative abundance (%)	With (+) or without (−) mucus
*Aphanocapsa*	32.8	+
*Planktolyngbya*	18.1	−
*Merismopedia*	12.7	+
*Aphanothece*	8.7	+
*Cyanodictyon*	2.5	+
Total	74.2	

### Life-history experiments

Two life-history experiments were performed: the first at the Tanzanian Fisheries Research Institute (TAFIRI) in Mwanza City (Fig. 1) and the second (a control experiment) at the Netherlands Institute of Ecology (NIOO-KNAW, Wageningen, the Netherlands). The first experiment was carried out to test the life-history effects of natural sestonic food (i.e. algae, bacteria, heterotrophic protozoans and detritus) collected in both the dry and rainy seasons from three different stations (Fig. 1) in Lake Victoria’s Mwanza Gulf, resulting in six season-station treatments. The second experiment used defined food of proven high quality as a control treatment. Measured life-history traits included size of newborn *Daphnia*, size of first adult instar (i.e. size at maturity), fecundity (number of eggs), age of first adult instar (i.e. age at maturity), and juvenile daily growth rate.

In both experiments clone DL15 of *D. lumholtzi* was used, which was collected on 16 August 2010 from Nyegezi Bay, close to the research facility (Fig. 1). Prior to the start of experiments animals were adapted to laboratory conditions using either natural seston of Nyegezi Bay (experiment 1), or cultured *S. obliquus* (experiment 2). Each pre-culture was started with one female. The newborns from this female were collected and cultured until the third or fourth adult instar, which took 10–12 days. Experiments were started with broods produced by these third and fourth adult instars. We used newborns of similar size, not older than 12 hours. Daphnids were cultured individually in 100 mL glass tubes at a constant water temperature of 25 *±* 0.5°C, with dim light (10–15 Μmol·m^−2^ s^−1^) on surface of medium in tubes), at a light:dark regime of 12:12 h.

Individuals were cultured until the first adult instar and observations were carried out every 12 hours. Since the duration time of juvenile instars is ca. 50% of the adult instars (Vijverberg and Richter, 1982), maturity was defined based on instar duration and not on basis of the presence or absence of eggs in the brood chamber. For each of the seven treatments (six season-station combinations plus the control) 10–21 replicates were used. Each replicate took 7–9 days (duration from newborn to first adult instar).

In the first experiment at TAFIRI, daphnids were fed natural seston, collected by filtering lake water from each of the three stations in both the dry and rainy seasons, through a 104-μm sieve before use. In the second (control) experiment at NIOO, defined food of proven high quality for *Daphnia* species, i.e. log-phase uni-algal cells of *S. obliquus* grown in chemostats (Vijverberg, 1989) was used. Here, daily fresh food media were prepared. Prior to use, the algae were centrifuged and resuspended in 0.45 μm membrane filtered and aerated (> 24 hours) ground water. The algal biomass concentration was 1.0 mg C·L^−1^, estimated by measuring the optical density at 750 nm with a spectrophotometer.

Individual daphnids were examined with a dissection microscope, measured using a micrometer eyepiece to the nearest 0.03 mm, and fecundity was assessed by counting eggs or embryos in the brood chamber. Animals were measured from the top of their head, not including the helmet, to the base of the tail spine (Vijverberg and Richter, 1982). Size at maturity, fecundity, and juvenile growth rate are indicators of food quantity and quality: the higher, the better the food conditions (Gliwicz, 1990; Repka *et al.,* 1998). A delay in maturity (higher age at maturity) indicates low food concentration, poor food quality or a combination of both.

As mortality was low for all treatments i.e. 1–2% per day, which corresponds to the mortality rate found for “well-designed” cultures of *Daphnia galeata* (Vijverberg, 1989), registration of mortality was not included in the experimental protocol.

Before carbon analysis, individuals were first washed in deionized water in order to exclude external carbon sources, and then dried in tin cups. Newborns and first adult instars were dried in an oven for at least 48 hours at 60°C and later at NIOO (Netherlands) the carbon content was determined with a C-N analyzer (Elemental Analyzer, Eurovector, Pavia, Italy). Based on the carbon content of newborns and first adult instars and on the age at maturity, juvenile growth rate (G) was calculated according to Gliwicz (1990):


$$ G=\frac{\mathit{\ln}\left({C}_{Ad}\right)-\mathit{\ln}\left({C}_{Nb}\right)}{t} $$

where *C_Ad_* and *C_Nb_* are individual carbon contents of adults and newborns, and *t* is the age of the first adult instar in days. *C_Nb_* was not successfully measured during the rainy season and these values were reconstructed from a power regression of *C_Nb_* on newborn length from all other individuals resulting in ${C}_{Nb}=2.54s\cdot{L_{Nb}}^{2.397}$, where *L_Nb_* is the size of the newborn (mm).

### Data analysis

All statistical analyses were performed using R, version 4.3.3 (R Core Team, 2024). Chlorophyll content values and the five life-history parameters of *D. lumholtzi* fed on natural seston were tested for the effects of station, season, and their interaction, using ANOVA. Validity of statistical models was assessed through visual inspection of Q-Q plots. Differences among stations, seasons and, in case of the life-history parameters, controls were tested by performing *post hoc* simultaneous multiple comparisons using the package “multcomp” (Hothorn *et al.,* 2008). A principal component analysis (PCA) was conducted to identify patterns in the five life-history parameters of *D. lumholtzi* fed on natural seston across the control, seasons, and stations.

All life-history data can be found in the supplementary material (Table S2).

## RESULTS

### Phytoplankton biomass

**Fig. 2:**
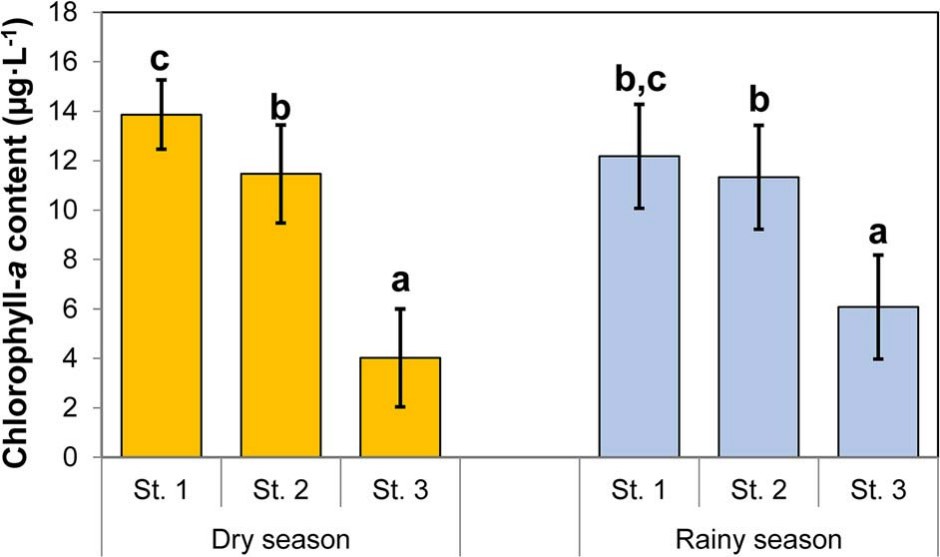
Mean chlorophyll *a* content (μg L^−1^) and their simultaneous confidence limits in Mwanza Gulf at sampling stations 1, 2 and 3 in August/September 2010 (dry season) and April 2011 (rainy season). Number of sampling dates per station per season: Dry season n = 4; Rainy season n = 5. Identical letters indicate non-significant differences (Tukey; *P* > 0.05). Based on data from Frank *et al.* (2023).

Mean phytoplankton biomass was measured as μg L^−1^ chlorophyll *a* content (Fig. 2). Chlorophyll concentrations were highly significantly affected by station and the interaction between station and season (*P* < 0.001). Season itself did not have a significant effect on chlorophyll concentrations (Table II). Chlorophyll content of Station 3 (mouth of the Mwanza Gulf in Lake Victoria) was much lower than in the other stations (29–53%: Fig. 2). Stations 1 and 2 were more similar and only significantly different in the dry season.

**Table II:** ANOVA analysis of the variations of chlorophyll a concentrations and life-history traits of Daphnia lumholtzi fed natural seston from Mwanza Gulf from sampling stations 1, 2 and 3 in August/September 2010 (dry season) and April 2011 (rainy season). Df = degrees of freedom; NS = non-significant

	Station			Season			Station × season		
	df	F	*p-*value	df	F	*p-*value	df	F	*p-*value
Chlorophyll concentration	3	130.5	<0.0001	1	0.03	0.86 (NS)	2	5.1	<0.01
*Life-history parameters*									
Size at maturity	3	122.2	<0.0001	1	80.75	<0.0001	2	10.0	<0.0001
Age at maturity	3	21.4	<0.0001	1	20.59	<0.0001	2	15.4	<0.0001
Fecundity	3	46.6	<0.0001	1	15.88	<0.0005	2	16.5	<0.0001
Size of newborn	3	4.7	<0.01	1	961.29	<0.0001	2	0.2	0.82 (NS)
Growth	3	32.1	<0.0001	1	17.24	<0.0001	2	9.6	<0.001

### Life-history experiments

**Fig. 3:**
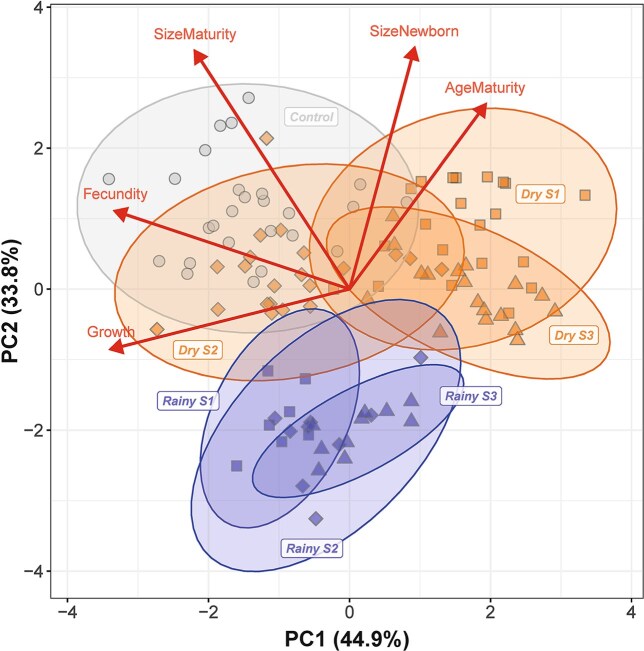
PCA of life-history parameters. Each data-point represents an observation. 95%-confidence ellipses are shown for each season (“Dry”and “Rainy”) and station (“S1”, “S2”, “S3”), and for the control treatment. Five life-history parameters are indicated by arrows.

The PCA summarized 78.7% of the variability in life-history traits of *D. lumholtzi* in the experiments (Fig. 3)*.* When considering the overall pattern of life-history parameters, *Daphnia* from the rainy season clearly differed from the dry season and the control treatment, while the differences between stations was much smaller in the rainy season than in the dry season. The control treatment overlapped mostly with station 2 during the dry season.

More detailed testing of the effects of station, season, their interactions and differences with the control treatment further specifies the PCA-patterns. We found a highly significant effect of station and season, and their interactions (*P* < 0.001) on all five life-history traits, except for the station-season interaction on the size of newborns (Table II).

**Fig. 4:**
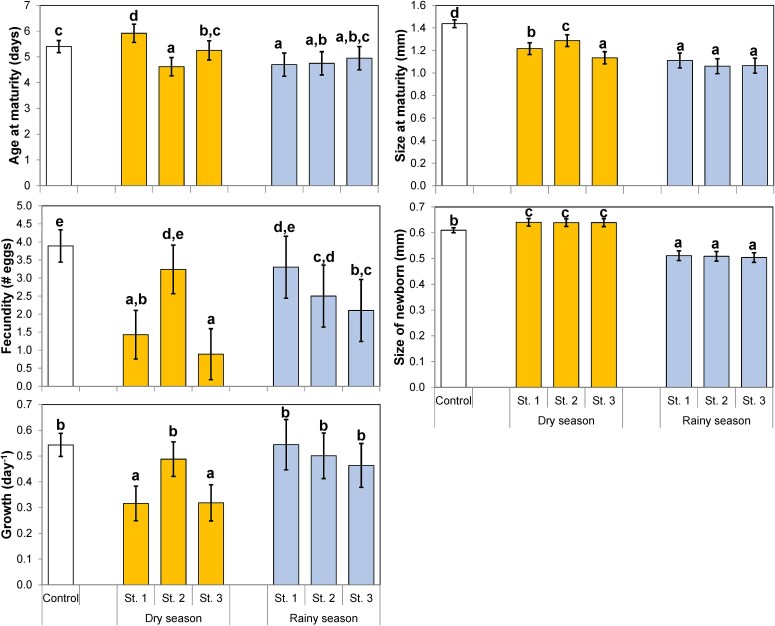
Means with simultaneous confidence intervals of life-history parameters of *Daphnia lumholtzi* fed on natural seston of the Mwanza Gulf from sampling stations 1, 2 and 3, respectively, in August/September 2010 (dry season) and April 2011 (rainy season) and fed on uni-algal cells of *Scenedesmus obliquus* grown in chemostats in a concentration of 1.0 mg C·L^−1^ (Control). Identical letters indicate non-significant differences (*P* > 0.05).

Size and age at maturity were generally larger in the dry season, resulting in larger newborns (Fig. 4). Fecundity in the dry season was however generally lower compared to the rainy season, especially at station 3, and juvenile growth rate showed a similar with both stations 1 and 3 showing significantly lower values than all growth rates in the rainy season (Fig. 4). Therefore, in general, food conditions appeared better in the rainy season. The control treatment was expected to be optimal and therefore have the highest values for all life-history parameters, but this was only the case for size at maturity and for fecundity (Fig. 4).

## DISCUSSION

Life-history traits of *D. lumholtzi* varied with seasons and stations when fed with natural seston from Lake Victoria, as well as with an (optimal) control treatment. As the control experiment was carried out at another laboratory than the other treatments it cannot be excluded that this has affected the results to some extent. However, as we carefully kept all laboratory circumstances, such as temperature and light regime as similar as possible, we may assume that this effect was only small.

In the rainy season *D. lumholtzi* grew and developed faster on seston than in the dry season. This is indicated by the generally smaller size of the newborns, smaller size at maturity, lower age at maturity, and higher somatic growth rate and fecundity as compared to the dry season (Fig. 4). The effect of station was more varied and usually more distinct in the dry season (Figs. 3, 4). This finding was contrary to our expectations, as algal biomass, measured as chlorophyll *a* concentration, was not significantly different between seasons, while it was consistently different between stations (Fig. 2). A potential explanation for this finding could be that there was a distinct difference in phytoplankton composition and algal densities between the two seasons. In the rainy season the abundance of small colonies of Cyanobacteria and filamentous algae dominated by *Planktolyngbya* spp. were substantially higher (Frank *et al.,* 2023: Table II, Figs. 3, 4). Apparently, chlorophyll content *per se* is a poor indicator of food availability and species composition of the phytoplankton community is of greater importance to explain zooplankton dynamics (Chang *et al.,* 2014). Also, growth rates were high in the rainy season and at station 2 in the dry season, i.e. similar to growth rates in the (optimal) control treatment. In addition, fecundity was quite high and not significantly different from the control treatment at station 1 in the dry season, and at station 2 in the rainy season.

It is not clear how it is possible that in the present life-history study *D. lumholtzi* was growing and developing so well compared to the optimal conditions of the control. There are a number of potential explanations that we explore here, although we did not directly investigate these, and evidence is circumstantial. First, as the abundance and dominance of inedible Cyanobacteria increase, the coupling between heterotrophic protozoans and zooplankton will become stronger (Riemann and Christoffersen, 1993; Pace *et al.,* 1998). Therefore, cladocerans may have the possibility of additional or alternative food sources. *D. lumholtzi* was possibly using non-algal food sources, such as the microbial food web (Picard and Lair, 2000). Secondly, several studies showed that zooplankton tolerance for toxic metabolites is highly variable among and within species via the rapid evolution of local adaptation (Hairston *et al.,* 1999; Tillmanns *et al.,* 2008; Kuster and Von Elert, 2013). It was shown that *Daphnia* genotypes isolated from eutrophic habitats are likely to be more tolerant to toxic Cyanobacteria than genotypes isolated from oligotrophic habitats (Hairston *et al.,* 2001; Sarnelle and Wilson, 2005). This could also be the case for our experimental cladoceran *D. lumholtzi*, which was isolated from the eutrophic Mwanza Gulf shortly before the start of the first life-history experiment.

Moreover, it was previously reported that daphnids may grow and develop well on Cyanobacteria and/or detritus of cyanobacterial origin with attached bacteria. For instance, Repka (1996) showed in life-history experiments that *Daphnia* was growing and reproducing well on a diet of pure cultured *Pseudanabaena limnetica*, although not as good as on a high-quality diet of uni-algal cells of *S. obliquus*. *Daphnia* also grew and reproduced well on natural seston from Tjeukemeer, a highly eutrophic lake in the Netherlands, which contained a high proportion of the Cyanobacteria *Planktothrix agardhii, Limnothrix redekei* en *Pseudanabaena limnetica* filaments (Boersma, 1995). Here, growth rate and fecundity of *Daphnia* were only slightly lower than when fed uni-algal cells of *S. obliquus.*

In addition, detritus, derived from the filamentous cyanobacterium *Pseudanabaena limnetica*, fed to *Daphnia* in a laboratory life-history experiment supported growth and development comparable to that of the uni-algal cells of the green alga *S. obliquus* (Repka *et al.,* 1998). Therefore, detritus from cyanobacterial origin can be a good-quality food for cladocerans in eutrophic lakes. Cornelissen *et al.* (2014) found a high rate of attenuation of photosynthetically available radiation in Mwanza Gulf compared to other eutrophic gulfs of Lake Victoria, which could only be partially explained by phytoplankton biomass. This strongly suggests the presence of high concentrations of suspended particulate detritus (Cornelissen *et al.,* 2014), which in combination with attached bacteria may serve as food for *Daphnia* (Boersma, 1995; Repka *et al.,* 1998). Apparently, algal food quality and toxic cyanobacterial metabolites are not limiting *D. lumholtzi* populations in Mwanza Gulf. The continued, and rather constant presence of low densities of (small-bodied) cladocerans in Mwanza Gulf since the 1950’s corroborates this (Rzóska, 1957  Akiyama et al., 1977, Waya and Mwambungu 2004, Suppl. Material Table S1).

Both eutrophication and predation play an important role in shaping the zooplankton community. Still, large-bodied cladocerans such as *D. lumholtzi* may remain in relatively low population densities by predation regardless of adequate food conditions. Gliwicz *et al.* (2010) found that cladoceran body size reflects vulnerability to fish predation with small-bodied cladocerans remaining in higher relative densities than large-bodied cladocerans under fish predation, regardless of food and reproduction levels. This may additionally explain the dominance of small-bodied cladocerans and low densities of large-bodied daphnids such as *D. lumholtzi* in Mwanza Gulf.

## CONCLUSION

Growth and development of *D. lumholtzi* on natural seston from Lake Victoria’s Mwanza Gulf were just as high, or only slightly lower than in a positive control treatment with proven high-quality food for *Daphnia* species, i.e. a laboratory-strain of the green alga *S. obliquus*. Although circumstantial, this suggests that Cyanobacteria and/or their detritus could have been better food than expected and that food quality is not limiting the growth of *D. lumholtzi* in *L. Victoria*.

## Supplementary Material

Supplementary_material_fbaf042

## Data Availability

All data underlying the life-history experiments of this study can be found in the supplementary data (Table S2).

## Notes

Corresponding Editor: Lars-Anders Hansson
