# Supplementary material for: Effects of food quantity and quality on the life history of Daphnia lumholtzi in Mwanza Gulf (Lake Victoria, Tanzania)
Source: J Plankton Res. 2025 Aug 30;47(5):fbaf042. doi: 10.1093/plankt/fbaf042 (PMC12397853; doi:10.1093/plankt/fbaf042)
Supplement: Supplementary_material_fbaf042 [file supplementary_material_fbaf042.docx]

**Supplementary material**

Table S1: Percentage composition of crustacean zooplankton taxa and cladoceran species in the Mwanza Gulf. Data was collected during the day at the three stations (Fig. 1) during three seasons (dry season, long and short rains) from 2009-2011. At each station, a pooled sample was collected by conducting 3 vertical hauls with a 150 µm mesh-sized conical plankton net. In total, 39 pooled samples were collected from 2009-2011. Samples were preserved in 5% formaldehyde and later examined with a microscope.

| Taxa | Percentage |
| --- | --- |
| Crustacean zooplankton | |
| Cyclopoida | 78.4 |
| Calanoida | 16.6 |
| Cladocera | 5.1 |
| Cladoceran species |  |
| *Moina micrura* | 45.0 |
| *Bosmina longirostris* | 28.9 |
| *Diaphanosoma* spp. | 14.1 |
| *Daphnia lumholtzi* | 5.4 |
| *Ceriodaphnia cornuta* | 4.7 |
| *Daphnia laevis* | 1.9 |
| *Chydorus* spp. | 0.1 |

Table S2: Life-history parameters of all individuals from the three experiments.

| **Individual** | **Season / Control** | **Station** | **Size of newborn (mm)** | **Size at maturity (mm)** | **Age at maturity (day)** | **Fecundity (# eggs)** | **Carbon content of newborn (µg)** | **Carbon content of adult (µg)** |
| --- | --- | --- | --- | --- | --- | --- | --- | --- |
| 1 | Rainy | S1 | 0.500 | 1.11 | 4.25 | 3 |  | 7.12 |
| 2 | Rainy | S1 | 0.540 | 1.16 | 4.75 | 3 |  | 6.05 |
| 3 | Rainy | S1 | 0.500 | 1.14 | 4.75 | 3 |  | 7.31 |
| 4 | Rainy | S1 | 0.510 | 1.14 | 4.75 | 3 |  | 6.00 |
| 5 | Rainy | S1 | 0.500 | 1.09 | 4.75 | 3 |  | 4.89 |
| 6 | Rainy | S1 | 0.500 | 1.09 | 4.75 | 3 |  | 7.17 |
| 7 | Rainy | S1 | 0.510 | 1.07 | 4.75 | 3 |  |  |
| 8 | Rainy | S1 | 0.540 | 1.16 | 4.75 | 4 |  | 6.77 |
| 9 | Rainy | S1 | 0.520 | 1.06 | 4.75 | 4 |  |  |
| 10 | Rainy | S1 | 0.510 | 1.10 | 4.75 | 4 |  |  |
| 11 | Rainy | S2 | 0.510 | 1.03 | 4.25 | 2 |  | 5.95 |
| 12 | Rainy | S2 | 0.500 | 0.94 | 4.25 | 2 |  | 5.61 |
| 13 | Rainy | S2 | 0.510 | 1.10 | 4.75 | 3 |  | 5.05 |
| 14 | Rainy | S2 | 0.510 | 1.09 | 4.75 | 3 |  | 6.96 |
| 15 | Rainy | S2 | 0.500 | 1.09 | 4.75 | 2 |  | 4.82 |
| 16 | Rainy | S2 | 0.510 | 1.09 | 4.75 | 3 |  | 5.39 |
| 17 | Rainy | S2 | 0.540 | 1.05 | 4.75 | 2 |  | 4.79 |
| 18 | Rainy | S2 | 0.530 | 1.09 | 5.75 | 2 |  | 4.93 |
| 19 | Rainy | S2 | 0.500 | 1.11 | 4.75 | 4 |  | 4.86 |
| 20 | Rainy | S2 | 0.500 | 1.04 | 4.75 | 2 |  |  |
| 21 | Rainy | S3 | 0.500 | 1.09 | 4.25 | 2 |  | 3.86 |
| 22 | Rainy | S3 | 0.510 | 1.06 | 4.75 | 2 |  | 5.05 |
| 23 | Rainy | S3 | 0.520 | 1.04 | 5.25 | 2 |  | 5.43 |
| 24 | Rainy | S3 | 0.480 | 1.11 | 5.25 | 2 |  | 4.93 |
| 25 | Rainy | S3 | 0.500 | 1.04 | 5.25 | 2 |  | 3.06 |
| 26 | Rainy | S3 | 0.480 | 1.06 | 5.75 | 2 |  | 4.24 |
| 27 | Rainy | S3 | 0.540 | 1.06 | 4.75 | 2 |  | 5.09 |
| 28 | Rainy | S3 | 0.510 | 1.09 | 4.75 | 3 |  | 5.05 |
| 29 | Rainy | S3 | 0.500 | 1.09 | 4.75 | 2 |  | 6.20 |
| 30 | Rainy | S3 | 0.510 | 1.01 | 4.75 | 2 |  | 5.90 |
| 31 | Dry | S1 | 0.650 | 1.24 | 5.75 | 2 | 0.57 | 4.25 |
| 32 | Dry | S1 | 0.660 | 1.16 | 6.75 | 0 | 1.15 | 4.97 |
| 33 | Dry | S1 | 0.650 | 1.21 | 5.25 | 2 | 1.11 | 4.31 |
| 34 | Dry | S1 | 0.630 | 1.32 | 6.25 | 2 | 0.73 | 3.69 |
| 35 | Dry | S1 | 0.630 | 1.24 | 6.75 | 2 | 0.90 | 5.50 |
| 36 | Dry | S1 | 0.630 | 1.27 | 4.75 | 2 | 0.87 | 8.85 |
| 37 | Dry | S1 | 0.650 | 1.29 | 6.25 | 2 | 0.45 | 6.17 |
| 38 | Dry | S1 | 0.660 | 1.27 | 6.25 | 2 | 1.12 | 8.16 |
| 39 | Dry | S1 | 0.660 | 1.18 | 4.75 | 2 | 0.61 | 4.43 |
| 40 | Dry | S1 | 0.650 | 1.31 | 6.25 | 2 | 0.67 | 7.21 |
| 41 | Dry | S1 | 0.650 | 1.27 | 5.25 | 2 | 0.76 | 6.20 |
| 42 | Dry | S1 | 0.610 | 1.16 | 6.25 | 1 | 0.70 | 6.11 |
| 43 | Dry | S1 | 0.660 | 1.18 | 6.25 | 2 | 0.89 | 7.40 |
| 44 | Dry | S1 | 0.660 | 1.16 | 6.25 | 1 | 0.60 | 6.72 |
| 45 | Dry | S1 | 0.650 | 1.24 | 6.25 | 2 | 1.81 | 5.13 |
| 46 | Dry | S1 | 0.630 | 1.18 | 6.25 | 2 | 1.24 | 4.88 |
| 47 | Dry | S1 | 0.620 | 1.16 | 5.25 | 2 | 1.62 | 5.86 |
| 48 | Dry | S1 | 0.630 | 1.10 | 5.75 | 0 | 1.18 | 5.86 |
| 49 | Dry | S1 | 0.610 | 1.10 | 5.75 | 0 | 1.19 | 7.93 |
| 50 | Dry | S1 | 0.630 | 1.38 | 6.25 | 0 | 1.20 | 6.29 |
| 51 | Dry | S1 | 0.650 | 1.16 | 5.75 | 0 | 1.21 | 6.07 |
| 52 | Dry | S2 | 0.610 | 1.32 | 4.04 | 4 | 0.57 | 10.14 |
| 53 | Dry | S2 | 0.650 | 1.27 | 4.13 | 3 | 1.15 | 10.23 |
| 54 | Dry | S2 | 0.630 | 1.27 | 4.13 | 3 | 1.11 | 9.38 |
| 55 | Dry | S2 | 0.630 | 1.27 | 4.13 | 4 | 0.73 | 8.24 |
| 56 | Dry | S2 | 0.650 | 1.24 | 4.13 | 3 | 0.90 | 7.27 |
| 57 | Dry | S2 | 0.660 | 1.24 | 4.13 | 3 | 0.87 | 8.82 |
| 58 | Dry | S2 | 0.650 | 1.32 | 4.75 | 3 | 0.45 | 9.56 |
| 59 | Dry | S2 | 0.630 | 1.32 | 4.75 | 2 | 1.12 | 8.59 |
| 60 | Dry | S2 | 0.650 | 1.27 | 4.75 | 2 | 0.61 | 9.04 |
| 61 | Dry | S2 | 0.660 | 1.32 | 4.75 | 4 | 0.67 | 7.78 |
| 62 | Dry | S2 | 0.630 | 1.32 | 4.75 | 4 | 0.76 | 9.40 |
| 63 | Dry | S2 | 0.610 | 1.24 | 4.75 | 3 | 0.70 | 6.49 |
| 64 | Dry | S2 | 0.650 | 1.46 | 5.75 | 5 | 0.89 | 10.92 |
| 65 | Dry | S2 | 0.660 | 1.32 | 4.25 | 4 | 0.60 | 7.37 |
| 66 | Dry | S2 | 0.620 | 1.32 | 4.25 | 4 | 1.81 | 6.84 |
| 67 | Dry | S2 | 0.630 | 1.24 | 5.25 | 2 | 1.24 | 7.09 |
| 68 | Dry | S2 | 0.620 | 1.21 | 5.25 | 2 | 1.62 | 5.83 |
| 69 | Dry | S2 | 0.650 | 1.29 | 4.75 | 2 | 1.74 | 7.08 |
| 70 | Dry | S2 | 0.630 | 1.29 | 4.75 | 4 | 1.12 | 6.96 |
| 71 | Dry | S2 | 0.660 | 1.32 | 4.75 | 4 | 0.78 | 6.78 |
| 72 | Dry | S2 | 0.660 | 1.21 | 4.75 | 3 | 0.43 | 8.31 |
| 73 | Dry | S3 | 0.630 | 1.21 | 4.75 | 2 | 0.57 | 4.15 |
| 74 | Dry | S3 | 0.630 | 1.24 | 4.75 | 2 | 1.15 | 3.31 |
| 75 | Dry | S3 | 0.670 | 1.24 | 5.25 | 3 | 1.11 | 5.94 |
| 76 | Dry | S3 | 0.630 | 1.24 | 5.25 | 3 | 0.73 | 3.32 |
| 77 | Dry | S3 | 0.650 | 1.16 | 5.25 | 2 | 0.90 | 5.70 |
| 78 | Dry | S3 | 0.620 | 1.14 | 5.75 | 2 | 0.87 | 4.01 |
| 79 | Dry | S3 | 0.610 | 1.16 | 5.75 | 0 | 0.45 | 4.29 |
| 80 | Dry | S3 | 0.620 | 1.16 | 5.25 | 2 | 1.12 | 3.31 |
| 81 | Dry | S3 | 0.650 | 1.07 | 5.25 | 0 | 0.61 | 3.58 |
| 82 | Dry | S3 | 0.660 | 0.99 | 5.25 | 0 | 0.67 | 4.18 |
| 83 | Dry | S3 | 0.620 | 1.05 | 5.25 | 0 | 0.76 | 3.42 |
| 84 | Dry | S3 | 0.650 | 1.07 | 5.25 | 0 | 0.70 | 5.22 |
| 85 | Dry | S3 | 0.650 | 1.24 | 5.25 | 0 | 0.89 | 6.30 |
| 86 | Dry | S3 | 0.610 | 1.16 | 5.25 | 0 | 0.60 | 6.32 |
| 87 | Dry | S3 | 0.660 | 1.07 | 5.25 | 0 | 1.81 | 5.84 |
| 88 | Dry | S3 | 0.660 | 1.07 | 5.25 | 0 | 1.24 | 9.99 |
| 89 | Dry | S3 | 0.650 | 1.05 | 5.25 | 0 | 1.62 | 4.43 |
| 90 | Dry | S3 | 0.660 | 1.13 | 5.25 | 0 | 1.74 | 7.25 |
| 91 | Control |  | 0.590 | 1.29 | 5.75 | 3 | 0.57 | 7.95 |
| 92 | Control |  | 0.590 | 1.32 | 5.75 | 4 | 0.57 | 9.38 |
| 93 | Control |  | 0.590 | 1.50 | 5.75 | 3 | 0.64 | 16.38 |
| 94 | Control |  | 0.590 | 1.50 | 5.75 | 4 | 0.64 | 15.12 |
| 95 | Control |  | 0.590 | 1.35 | 6.25 | 3 | 0.71 | 10.71 |
| 96 | Control |  | 0.590 | 1.47 | 5.25 | 4 | 0.71 | 16.54 |
| 97 | Control |  | 0.620 | 1.53 | 6.25 | 5 | 0.69 | 20.23 |
| 98 | Control |  | 0.620 | 1.35 | 5.25 | 4 | 0.69 | 9.44 |
| 99 | Control |  | 0.620 | 1.35 | 6.25 | 3 | 0.72 | 10.66 |
| 100 | Control |  | 0.620 | 1.26 | 6.25 | 3 | 0.72 | 6.08 |
| 101 | Control |  | 0.650 | 1.38 | 5.25 | 3 | 0.87 | 14.27 |
| 102 | Control |  | 0.650 | 1.53 | 5.75 | 5 | 0.85 | 18.55 |
| 103 | Control |  | 0.650 | 1.53 | 6.25 | 5 | 0.77 | 18.34 |
| 104 | Control |  | 0.650 | 1.41 | 5.25 | 4 | 0.72 | 13.66 |
| 105 | Control |  | 0.590 | 1.44 | 4.75 | 4 | 0.72 | 13.45 |
| 106 | Control |  | 0.590 | 1.41 | 4.75 | 4 | 0.77 | 16.17 |
| 107 | Control |  | 0.590 | 1.53 | 4.75 | 3 |  |  |
| 108 | Control |  | 0.590 | 1.47 | 4.75 | 4 | 0.56 | 14.94 |
| 109 | Control |  | 0.590 | 1.59 | 5.25 | 6 | 0.56 | 17.42 |
| 110 | Control |  | 0.590 | 1.44 | 4.75 | 2 | 0.77 | 13.45 |
| 111 | Control |  | 0.620 | 1.53 | 5.75 | 5 | 0.77 | 19.48 |
| 112 | Control |  | 0.620 | 1.44 | 5.25 | 4 | 0.73 | 14.12 |
| 113 | Control |  | 0.620 | 1.35 | 4.75 | 3 | 0.73 | 10.38 |
| 114 | Control |  | 0.620 | 1.47 | 5.25 | 4 | 0.80 | 8.83 |
| 115 | Control |  | 0.620 | 1.53 | 5.25 | 5 | 0.80 | 18.34 |
| 116 | Control |  | 0.620 | 1.41 | 4.75 | 4 | 0.96 | 12.10 |
| 117 | Control |  | 0.650 | 1.41 | 4.75 | 4 | 0.96 | 16.43 |
